# To What Extent do Social Determinants of Health Modulate Presentation, ITU Admission and Outcomes among Patients with SARS-COV-2 Infection? An Exploration of Household Overcrowding, Air Pollution, Housing Quality, Ethnicity, Comorbidities and Frailty

**Published:** 2021-02-25

**Authors:** MA Soltan, LE Crowley, CR Melville, J Varney, S Cassidy, R Mahida, FS Grudzinska, D Parekh, DP Dosanjh, DR Thickett

**Affiliations:** 1 University Hospitals Birmingham Foundation NHS trust, Queen Elizabeth Hospital Birmingham, Birmingham, UK; 2 Birmingham Acute Care Research, Institute of Inflammation and Ageing, University of Birmingham, Birmingham, UK; 3 Department of Medical Sciences, University of Manchester, Manchester, UK; 4 Department of Public Health, Birmingham City Council, Birmingham, UK

**Keywords:** COVID19, BAME, Index of multiple deprivation, Birmingham city, Air pollution, Household overcrowding, Housing quality, Multimorbidities, Frailty, Radiological multi-lobar pneumonia

## Abstract

**Background:**

Internationally, researchers have called for evidence to support tackling health inequalities during the severe acute respiratory syndrome coronavirus 2 (COVID19) pandemic. Despite the 2020 Marmot review highlighting growing health gaps between wealthy and deprived areas, studies have not explored social determinants of health (ethnicity, frailty, comorbidities, household overcrowding, housing quality, air pollution) as modulators of presentation, intensive care unit (ITU) admissions and outcomes among COVID19 patients. There is an urgent need for studies examining social determinants of health including socioenvironmental risk factors in urban areas to inform the national and international landscape.

**Methods:**

An in-depth retrospective cohort study of 408 hospitalized COVID19 patients admitted to the Queen Elizabeth Hospital, Birmingham was conducted. Quantitative data analyses including a two-step cluster analysis were applied to explore the role of social determinants of health as modulators of presentation, ITU admission and outcomes.

**Results:**

Patients admitted from highest Living Environment deprivation indices were at increased risk of presenting with multi-lobar pneumonia and, in turn, ITU admission whilst patients admitted from highest Barriers to Housing and Services (BHS) deprivation Indies were at increased risk of ITU admission. Black, Asian and Minority Ethnic (BAME) patients were more likely, than Caucasians, to be admitted from regions of highest Living Environment and BHS deprivation, present with multi-lobar pneumonia and require ITU admission.

**Conclusion:**

Household overcrowding deprivation and presentation with multi-lobar pneumonia are potential modulators of ITU admission. Air pollution and housing quality deprivation are potential modulators of presentation with multi-lobar pneumonia. BAME patients are demographically at increased risk of exposure to household overcrowding, air pollution and housing quality deprivation, are more likely to present with multi-lobar pneumonia and require ITU admission. Irrespective of deprivation, consideration of the Charlson Comorbidity Score and the Clinical Frailty Score supports clinicians in stratifying high risk patients.

## Background

Globally, researchers have described social determinants of health pre-COVID19 pandemic [1] and among COVID19 patients [2] with several calls for evidence for tackling health inequalities during the COVID19 outbreak [3]. National UK data published by the Office for National Statistics (ONS) suggests that patients in regions of highest deprivation according to the overall Index of Multiple Deprivation Score (IMDS) are twice as likely to die of COVID19 than of other causes [4]. ONS data also suggests that patients from the city of Birmingham (UK) are twice as likely to die of COVID19 than the national average between the 1st of March 2020 and the 17th April 2020 [4]. The IMDS includes seven sub-domains of deprivation: Crime, Education, Health and disability, Income, Employment, Barriers to Housing and Services (BHS) and Living Environment. The BHS deprivation index includes an indicator for household overcrowding whilst the living environment deprivation index includes indicators for household quality and air pollution.

Studies have not focused thus far on exploring the roles of specific social determinants of health (ethnicity, frailty, comorbidities, household overcrowding, housing quality, air pollution) on presentation, ITU admissions and outcomes among COVID19 positive patients. Furthermore, despite the 2020 Marmot review highlighting growing health gaps between wealthy and deprived areas [1], studies have not explored these factors at the city level which is essential in informing the national and international picture. Birmingham is the 2nd largest city and the 3rd most deprived city in the United Kingdom with a diverse population, 40.04% BAME, and the full range of deprivation indices [5] making it particularly amenable to studying the roles of social determinants of health as modulators of outcomes among COVID19 positive patients. Studies have highlighted that the worst air pollution levels nationally are seen in ethnically diverse neighborhoods, with a high population of patients of BAME ethnicity [6] and UK Government statistics show that BAME households are more overcrowded with 30% of overcrowded households being of Asian ethnicity, 15% of Black ethnicity, 3% of Mixed ethnicity and 2% Caucasian ethnicity [7].

This study explores the role of social determinants of health, including socioenvironmental risk factors, as modulators of presentation, ITU admission and outcomes among COVID19 positive patients at the city level. A greater understanding of these factors will support front-line clinicians in risk stratifying patients and identifying the index of suspicion for care as well as informing wider pandemic strategic planning.

## Methods

### Design and setting

An in-depth cohort study of patients admitted to the Queen Elizabeth Hospital, Birmingham was performed to explore the role of social determinants of health as modulators of ITU admissions and outcomes among hospitalized COVID19 positive patients.

### Patient population

Patients (>16 years old) with confirmed COVID19 infection from the 1st March 2020 until 13th April 2020 were included. The COVID19 infection diagnosis was based upon PCR analysis of a combined nose and throat swab in accordance with Public Health England guidance.

**Figure 1:**
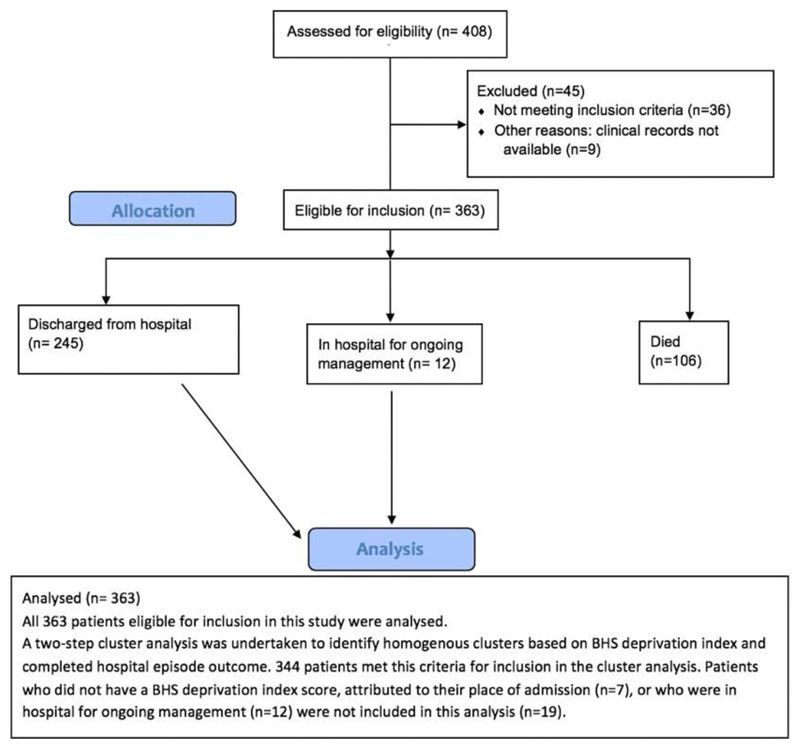
Consort flow diagram of patient eligibility for the study.

Four hundred and eight patients were assessed for eligibility for inclusion into this study. A CONSORT flow diagram of study participants is shown in Figure 1. Patients were excluded (n=45) on account of either having not met the inclusion criteria (n=36) or clinical records being unavailable (n=9). Patients eligible for inclusion (n=363) were analysed in this paper. A two-step cluster analysis was undertaken to identify homogenous clusters based on BHS deprivation index and completed hospitalised episode outcome (n=344). Patients who did not have a BHS deprivation score attributed to their place of admission (n=7), or who were in hospital for ongoing management (n=12) were not included in this analysis.

### Patient management

Patients were admitted and treated initially according to British Thoracic Society (BTS) guidelines for COVID19 community acquired pneumonia with antibiotics, fluids and controlled oxygen where appropriate. The hospital’s local antibiotic policy uses CURB-65 to risk stratify community acquired pneumonia patients. Trust infection prevention measures were followed.

All suspected COVID19 infected patients had a decision about escalation to critical care and discussion in relation to resuscitation status at their first review after admission (typically in less than 4 hours due to the introduction of resident consultants during the pandemic).

Patients who were for critical care escalation were reviewed by the critical care assessment team if they had an altered GCS, persistently low systolic blood pressure (<90 mmgHg), respiratory acidosis (pH<7.2) or were unable to maintain their target saturations or had a respiratory rate >30 breaths per minute despite receiving a fractional inspired oxygen (FiO2) of ≥ 0.5. If deemed appropriate, patients were intubated and transferred to critical care subsequently.

At the beginning of the pandemic, the trust introduced a rapid review Chest X-ray reporting service staffed by Consultant radiologists to ensure Chest X-rays were reported within 12 hours of being undertaken. Chest X-Rays were reviewed again by a Professor of Respiratory Medicine (DT) for the purposes of this study.

All patients were prescribed their regular medications for existing medical conditions whilst in hospital unless a medication was contraindicated for clinical reasons in which case it was paused temporarily until safe to resume. All patients received 40 mg subcutaneous enoxaparin as venous thromboembolic disease prophylaxis daily, unless it was contraindicated, as per our hospital policy.

### Data collection and scoring analysis

The hospital informatics system records each patient’s: demographics (ethnicity, age, place of admission, postcode), medical records (admission review, clinical assessments, escalation decisions, past medical history, comorbidities, management), clinical metrics (imaging), information about whether a patient was admitted to ITU and completed hospitalised episode outcomes (discharge or death).

#### Index of Multiple Deprivation Score (IMDS)

The IMDS incorporates 7 sub-domains of deprivation weighted as indicated: Crime (9.3%), Education (13.5%), Health and disability (13.5%), Income (22.5%), Employment (22.5%), Barriers to Housing and Services (BHS) (9.3%) and Living Environment (9.3%). The BHS sub-domain contains a sub-index with an indicator for household overcrowding. The Living Environment sub-domain contains two sub-indices with indicators for housing quality and air pollution. The IMD categorises deprivation metrics by postcode on a scale of 1 to 10 (most to least deprived centiles nationally) [8]. Detailed descriptions of the IMDS and its constituent sub- domains are published by the UK Ministry of Housing, Communities and Local Government Department [9].

#### Charlson Comorbidity Score (CCI)

CCI is a validated tool quantifying comorbidity burden and corresponding 1 year mortality risk [10].

#### Clinical frailty scale

NICE guidelines recommend that physicians use the Clinical Frailty Score, as measured by the Clinical frailty Scale, available from the NHS Specialized Clinical Frailty Network, when assessing adults for frailty irrespective of COVID19 status [11]. The Clinical Frailty Scale is a globally utilised and validated measure of frailty based on clinical judgement [12].

### CURB 65

Severity of presentation on admission was assessed using the CURB65 score in accordance with the British Thoracic Society Guidelines [13]. The CURB65 score, which is a simple, six point score based on confusion, urea, RR, BP and age is a validated score for the assessment of the severity of pneumonia on presentation [14].

### Statistical analysis

Baseline characteristics were presented as mean (standard deviation) for continuous variables and proportions were calculated for categorical data. Normality of distributions for quantitative variables was assessed by the Shapiro-Wilk test. For categorial variables with non-parametric distribution, Fisher’s exact test for comparison between two groups and Pearson’s Chi-squared test was used for comparisons between more than two groups. For ordinal variables with non-parametric distribution, Mann Whitney U test was used for comparisons between two groups and Kruskall Wallis was used for comparisons between more than two groups. Data is presented as a Median (IQR) for non-parametric data. To quantify an association between two variables with non-parametric distribution, Spearman’s correlation was used. Statistical analysis was carried out using IBM SPSS Statistics for MAC V.24 and Prism 8.

### Cluster analyses

Two-step cluster analysis is an exploratory analysis of a sample to identify homogenous groups of cases based on the distribution of the input variables using log-likelihood to model distances between variables. Cluster analysis identifies groupings by running pre-clustering first and then by hierarchical methods. This technique can detect latent relationships within a complex dataset between patients with multiple distinct characteristics. It is appropriate for continuous, ordinal and categorical data sets larger than 200 data points [15,16]. The number of clusters was determined automatically following the Bayesian Information criterion (BIC) using IBM SPSS Statistics for MAC V.24. When clusters had been identified within the samples, group comparisons were performed. Descriptive statistics were used to describe the data by clusters. For categorical and ordinal data, the X2 and Kruskall Wallis tests respectively were used to examine any significant differences between clusters. The post-hoc Bonferroni correction was applied for multiple group comparisons and all results were considered significant at p<0.05.

## Results

### Participant characteristics

**Figure 2:**
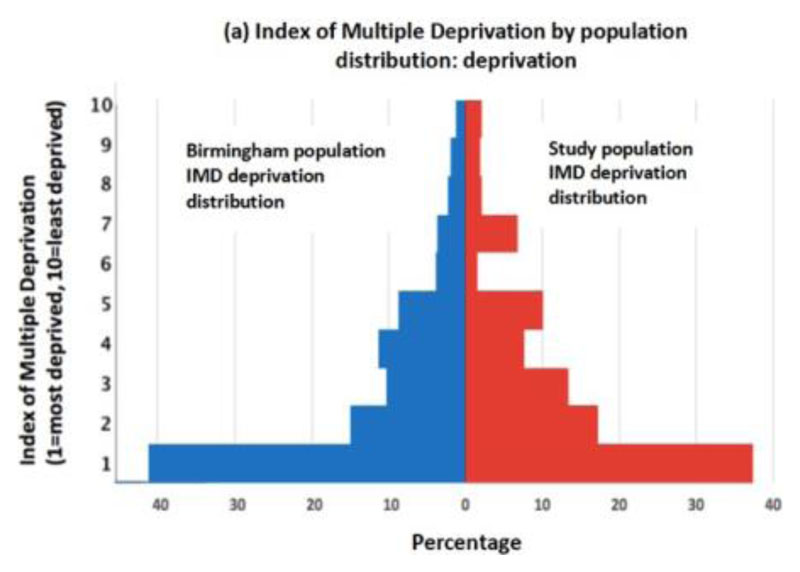

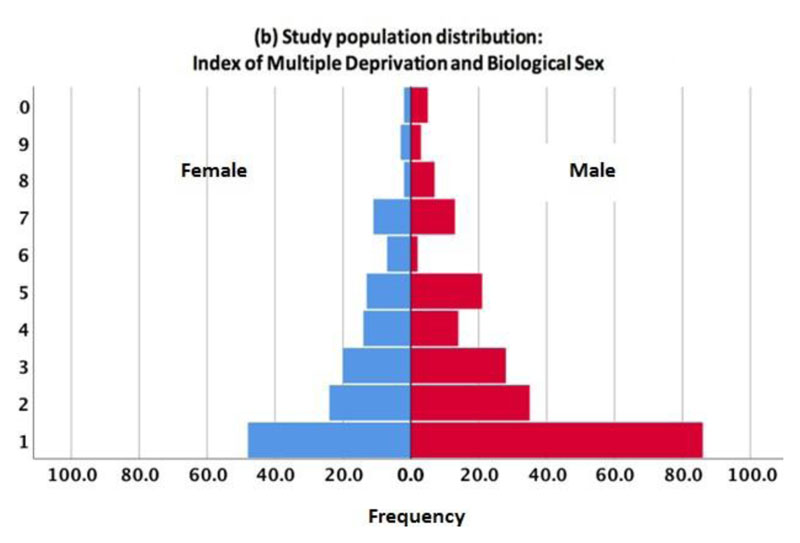

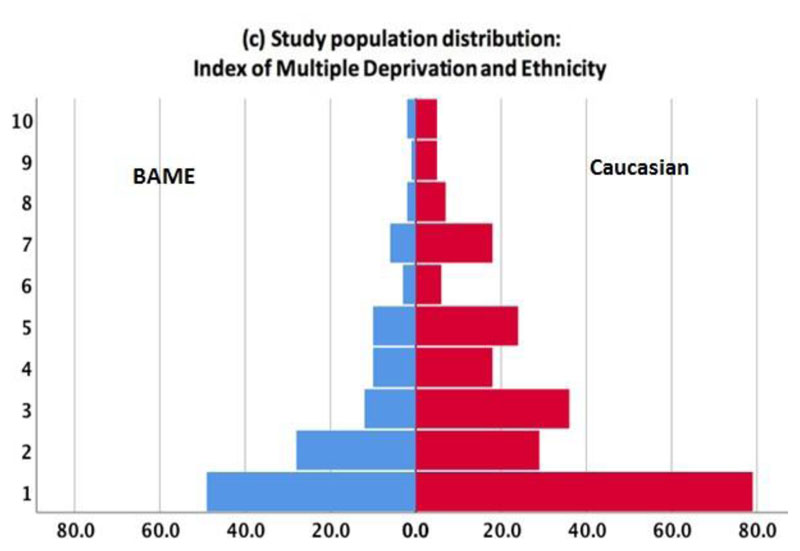

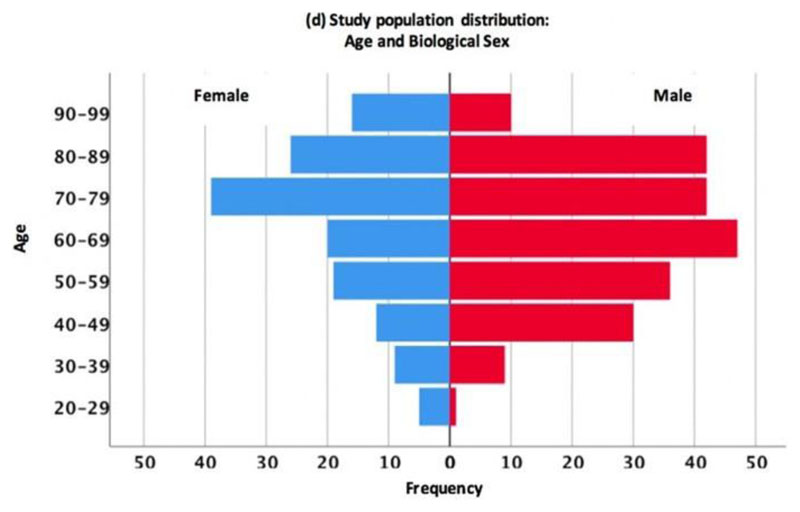

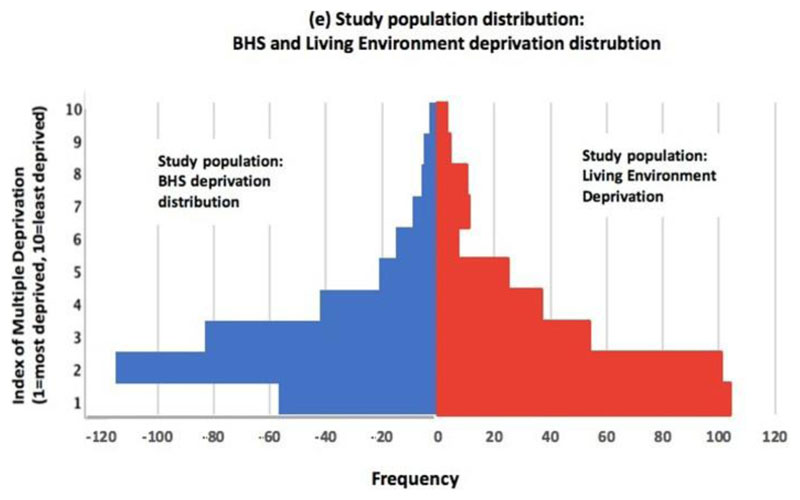
(a): Index of Multiple Deprivation by population distribution: study population in relation to the corresponding regional population. (b): Study population distribution: Index of multiple deprivation and biological sex. (c): Study population distribution: Index of multiple deprivation and ethnicity. (d): Study population distribution: Age and biological sex. (e): Study population distribution: BHS and living environment deprivation distribution.

**Figure 3:**
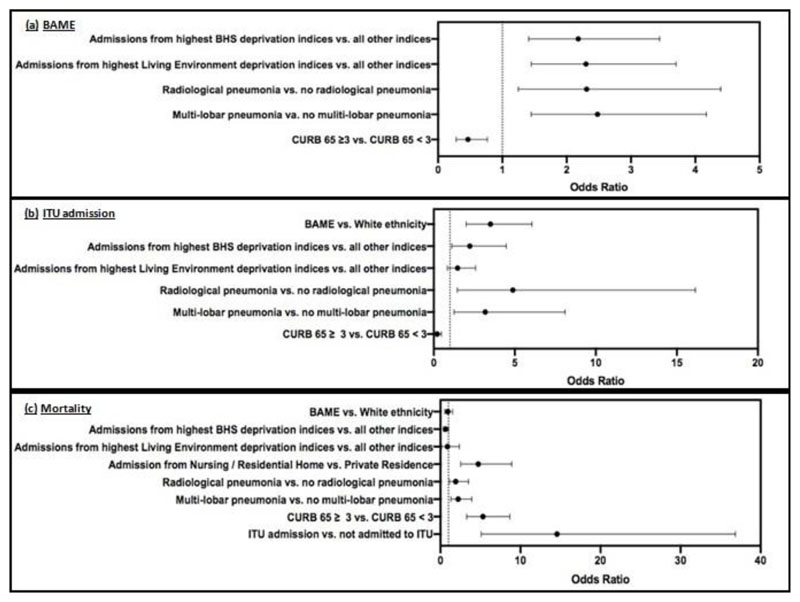
(a) **Odds ratios of BAME patients:** admission from regions of highest BHS deprivation indices (indices 1 and 2), admission from regions of highest Living Environment deprivation (indices 1 and 2), presentation with radiological pneumonia, presentation with radiological multi-lobar pneumonia and severity (CURB 65 ≥ 3). (b) **Odds ratios of ITU admission among COVID19 positive patients:** BAME ethnicity, admission from regions of highest BHS deprivation (indices 1 and 2), admission from regions of highest Living Environment deprivation (indices 1 and 2) presentation with radiological pneumonia, presentation with radiological multi-lobar pneumonia and severity (CURB 65 ≥ 3). (c) **Odds ratios of mortality among COVID19 positive patients:** BAME ethnicity, admission from regions of highest BHS deprivation (indices 1 and 2), admission from regions of highest Living Environment deprivation (indices 1 and 2), admission from nursing/residential homes, presentation with radiological pneumonia, presentation with radiological multi-lobar pneumonia, severity (CURB 65 ≥ 3) and admission to ITU.

The study population is outlined in Table 1 and Figure 2. Males (59.8%) were hospitalised more than females (40.2%). The mean age of all patients in this study was 67. Patients of BAME ethnicity were younger (mean-61.85 (14.5)) whilst patients of Caucasian ethnicity were older (mean-69.5 (17.3)). Patients of BAME ethincity constituted 31.2% of admissions whilst patients of Caucasian ethnicity constituted 63.6% of admissions. The IMDS population distribution for admissions was proportionally similar to the local city population (Figure 2a) (17- 19). Patients admitted from the highest BHS deprivation indices, 1 and 2, constituted 47.4% of all admissions whilst patients admitted from the highest Living Environment deprivation indices, 1 and 2, constituted 42.4% of all admissions (Figure 2e). 60.2% of BAME patients were populated within the highest BHS deprivation indices, 1 and 2, in comparison to 40.9% of Caucasian patients. 69.9% of BAME patients were populated within the highest Living Environment deprivation indices, 1 and 2, in comparison to 50.2% of Caucasian patients Figure 3a). Patients hospitalised from nursing homes (n=47) were all admitted from the top five most deprived BHS deprivation indices.

Patients hospitalised from regions of highest BHS deprivation, indices 1 and 2, were more likely to be admitted to ITU (OR 2.22, 95% CI 1.111-4.469, p=0.030). Patients hospitalised from regions of highest Living Environment deprivation, indices 1 and 2, were more likely to present with radiological multi-lobar pneumonia (OR 1.923, 95% CI 1.195-3.039, p=0.006) in comparison with patients admitted from all other Living Environment deprivation indices. Patients presenting with radiological multi-lobar pneumonia were more likely to be admitted to ITU (OR 3.174, 95% CI 1.250-8.103, p=0.019) and die (OR 2.224, 95% CI 1.296-3.928, p=0.004).

COVID19 positive patients of BAME ethnicity were more likely to be: admitted from regions of highest BHS deprivation (OR 2.18, 95% CI 1.410-3.445, p<0.001), admitted from regions of highest Living Environment deprivation (OR 2.30, 95% CI 1.450-3.701, p<0.001), present with radiological pneumonia (OR 2.31 95% CI 1.249-4.394, p=0.008) and present with multi-lobar pneumonia (OR 2.480, 95% CI 1.446-4.172, p<0.001) than patients of Caucasian ethnicity (Figure 3a). Furthermore, COVID19 positive patients who were admitted to ITU were more likely to: be of BAME ethnicity (OR 3.5 95% CI 2.00-6.06, p<0.001), be admitted from regions of highest BHS deprivation (OR 2.22, 95% CI 1.111-4.469, p=0.030), present with radiological pneumonia (OR 4.880, 95% CI 1.452-16.14, p=0.008) and present with multi-lobar pneumonia (OR 3.174, 95% CI 1.250-8.103, P=0.019) than COVID19 positive patients who were not admitted to ITU (Figure 3b).

Moreover, COVID19 positive patients who died were more likely to: be admitted from a nursing/residential home (OR 4.729, 95% CI 2.533 – 8.922, p<0.001), present with radiological pneumonia (OR 4.880, 95% CI 1.452-16.14, p=0.008), present with multi-lobar pneumonia (OR 2.224, 95% CI 1.296-3.928, p=0.004), present with increased severity, CURB 65 ≥ 3, (OR 5.32, 95% CI 3.267-8.662, p<0.001), and have been admitted to ITU during the inpatient stay (OR 14.57, 95% CI 5.089-36.85, P<0.001) than patients who were discharged (Figure 3c).

**Figure 4:**
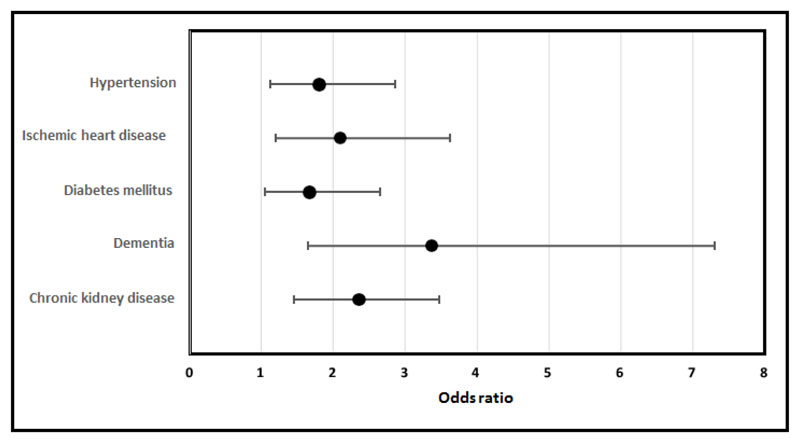
Odds ratios of mortality among hospitalised COVID19 positive patients with the respective underlying comorbidities (hypertension, ischaemic heart disease, diabetes mellitus, dementia, chronic kidney disease).

Individual comorbidities were significantly associated with increased risk of death among hospitalised COVID19 positive patients: hypertension (OR-1.806, 95% CI 1.129-2.864, p=0.014), ischaemic heart disease (OR-2.096, 95% CI 1.204-3.625, p=0.011), diabetes mellitus (OR 1.67 95%CI 1.046-2.650, p=0.037) dementia (OR-3.375, 95% CI 1.657-7.314, p=0.002) and chronic kidney disease (OR-2.36, 95% CI 1.452-3.841, p=0.001) (Figure 4). Charlson Comorbidity Scores were higher among COVID19 positive patients who died (Median 6 (IQR 4)) in comparison with patients who were discharged (Median 3 (IQR 5), p<0.001). Patients with higher Charlson Comorbidity Scores, presented with increased severity, CURB 65, (p<0.001). Charlson Comorbidity Index Scores were higher among patients of Caucasian ethnicity in comparison to patients of BAME ethnicity (Caucasian-median 5 (IQR 5), BAME-median- 3 (IQR 4.75), p=0.002). Patients of Caucasian ethnicity were more likely to present with increased severity on admission, CURB65 ≥ 3, (OR 2.15, 95% CI 1.306-3.39, p=0.0023) in comparison to patients of BAME ethnicity. Charlson Comorbidity Scores were also higher among patients admitted from nursing/residential homes in comparison to patients admitted from a private residence (Nursing/Residential home-Median 6 (IQR 3), Private residence-Median 4 (IQR 4), p=0.001). Patients admitted from nursing/residential homes were more likely to present with increased severity on admission, CURB65 ≥ 3, (OR 5.016, 95% CI 2.656-9.385, p<0.001) in comparison to patients admitted from a private residence.

Clinical Frailty Scores were higher among COVID19 positive patients who died (median-6.00 (IQR-3.50) in comparison to COVID19 positive patients who survived (median-3.00 (IQR-3.00), p<0.001). Patients admitted from Nursing/Residential homes had higher Clinical Frailty Scores than patients admitted from a private residence (Nursing/Residential home-Median 7 (IQR 1), Private residence-Median 3 (IQR 3), p<0.001). There was no statistical significance in Clinical Frailty Score between patients of BAME and Caucasian ethnicity. (BAME: median-3 (IQR-4), Caucasian: median-4 (IQR-4), p=0.188).

### Exploring the profiles of social determinants of health among patients clustered by BHS deprivation and outcome: two-step cluster analysis

A two-step cluster analysis was undertaken to identify homogenous clusters based on BHS deprivation, which contains an indicator for household overcrowding, and completed hospital episode outcome (n=344). Four distinct clusters emerged reflecting four statistically distinct groups (Table 2).

Cluster 1 is characterised by patients admitted from regions of highest BHS deprivation, indices 1-2, who died. Cluster 1 had relatively high proportions of: BAME patients, comorbidities among patients (chronic kidney disease, dementia, ischaemic heart disease, hypertension and type 2 diabetes mellitus) and admissions from nursing/residential homes in comparison to the corresponding proportions among all patients.

Cluster 2 is characterised by patients admitted from all other BHS deprivation indices who died. Cluster 2 had relatively high proportions of: Caucasian patients, comorbidities among patients (chronic kidney disease, dementia, ischaemic heart disease, hypertension and type 2 diabetes mellitus) and admissions from nursing/residential homes in comparison to the corresponding proportions among all patients.

Cluster 3 is characterised by patients admitted from regions of highest BHS deprivation, indices 1-2, who were discharged. Cluster 3 had a relatively high proportion of BAME patients but relatively lower proportions of: comorbidities among patients (chronic kidney disease, dementia, ischaemic heart disease, hypertension and type 2 diabetes mellitus) and admissions from nursing/residential homes in comparison to the corresponding proportions among all patients.

Cluster 4 is characterised by patients admitted from all other BHS deprivation indices who were discharged. Cluster 4 had a relatively high proportion of Caucasian patients (chronic kidney disease, dementia, ischaemic heart disease, hypertension and type 2 diabetes mellitus) and admissions from nursing/residential homes in comparison to the corresponding proportions among all patients.

**Figure 5:**
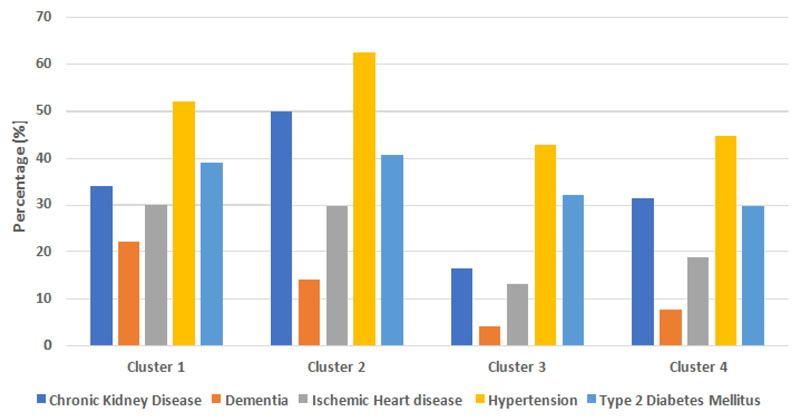
A graphical representation of comorbidities within groups of hospitalised COVID19 positive patients clustered by BHS deprivation and completed hospitalised episode outcome: Cluster 1 (patients admitted from regions of highest BHS deprivation who died), Cluster 2 (patients admitted from all other regions of BHS deprivation who died), Cluster 3 (patients admitted from regions of highest BHS deprivation who were discharged), Cluster 4 (patients admitted from all other regions of BHS deprivation who were discharged).

Table 2 shows the cluster membership profiles with respect to: age, gender, ethnicity, place of admission, comorbidities, Charlson Comorbidity Index and Clinical Frailty Score. Figure 5 shows a graphical representation of individual comorbidity profiles within each of the four clusters. Group comparisons were performed to examine the profiles of independent comorbidities, Charlson Comorbidity Scores and Clinical Frailty Scores.

### Examining comorbidities within clusters

Charlson Comorbidity Index scores for patients who died (clusters 1 and 2) were above the average for all patients (Median- 4.00, IQR-4.00) in regions of highest BHS deprivation (Cluster 1; Median-6.00, IQR-5.00) and in all other regions of BHS deprivation (Cluster 2; Median-6.00, IQR-3.00). Patients who were discharged from hospital had average or below average Charlson Comorbidity Index scores in both regions of highest BHS deprivation (Cluster 3; Median-2.00, IQR-4.00) and all other BHS deprivation (Cluster 4; Median-4.00, IQR-4.00). There was a statistically significant difference between the four cluster medians (p<0.001).

Among patients who died (clusters 1 and 2), irrespective of BHS deprivation, there was no statistically significant difference in comorbidities: chronic kidney disease (p=0.158), dementia (p=0.304), ischaemic heart disease (p=1.000), hypertension (p=0.413), type 2 diabetes mellitus (p=1.000). Among patients in the most deprived BHS regions (cluster 1 and 3), patients who died were more likely to have: chronic kidney disease (OR 2.619, 95% CI 1.180-5.697, p=0.0253), dementia (OR 6.525, 95% CI 2.021-18.16, p=0.001) and ischaemic heart disease (OR 2.813, 95% CI 1.242-6.611, p=0.028) but were not more likely to have hypertension (p=0.359) or type 2 diabetes mellitus (p=0.450). Figure 5 shows a graphical representation of comorbidities within each of the four clusters.

### Examining frailty within clusters

Clinical Frailty Scores for patients who died (clusters 1 and 2) were above the average for all patients (Median 4.00, IQR-4.00) in regions of highest BHS deprivation (Cluster 1; Median-6.00, IQR-4.00) and in all other regions of BHS deprivation (Cluster 2; Median-6, IQR-3.00). Patients who were discharged from hospital had average or below average Clinical Frailty Scores in regions of highest BHS deprivation (Cluster 3; Median-3.00, IQR-3.00) and all other BHS deprivation (Cluster 4; Median-4.00, IQR 4.00). There was a statistically significant difference between the four cluster medians (p<0.001).

## Discussion

In the present study we show that Living Environment deprivation and BHS deprivation modulate presentation with multilobar pneumonia and ITU admission respectively whilst comorbidities and frailty modulate mortality, irrespective of deprivation. Furthermore, this study finds that COVID19 positive patients of BAME ethnicity were more likely to be admitted from regions of highest Living Environment deprivation than Caucasians. This is likely to explain the higher proportion of BAME patients presenting with radiological pneumonia and multi-lobar pneumonia in comparison to Caucasians; this is despite BAME patients presenting younger and with lower Charlson Comorbidity Scores. Moreover, COVID19 positive patients admitted from nursing/residential homes were admitted from regions of high BHS deprivation and were more likely to present with higher Charlson comorbidity scores, higher Clinical Frailty Scores and die in comparison with COVID19 positive patients admitted from a private residence.

With respect to the reporting of deprivation, this study highlights that deprivation is a broad term encompassing different sub-indices (Income, Employment, Education, Crime, Living Environment, BHS, Health and disability). The overall IMDS is a non-specific measure for exploring the role of individual social determinants of health as modulators of outcomes among COVID19 positive patients. Rather than relying on the overall IMDS, this study is the first of its kind to explore the role of social determinants of health including individual IMDS sub-domains with indicators for specific socioenvironmental deprivation forms (BHS deprivation, living environment) as modulators of COVID19 positive patient admissions to ITU and outcomes at the city level which is essential in informing the national and international landscape.

This study presents a finding that Living Environment deprivation, which includes indicators for household quality and air pollution, modulates presentation with multi-lobar pneumonia among COVID19 positive patients which, in turn, increases the risk of a severe adverse event requiring admission to ITU. It is well established that exposure to air pollutants is associated with an increased incidence of pneumonia [20] yet studies thus far have not explored air pollutants as modulators of ITU admissions and outcomes among COVID19 positive patients. It is already established that the worst air pollution levels are seen in ethnically diverse neighbourhoods, with a high population of patients of BAME ethnicity [6]. This study finds that COVID19 positive patients of BAME ethnicity were more likely to be admitted from regions of highest living environment deprivation than patients of Caucasian ethnicity. This is likely to explain the increased presentation with radiological pneumonia and multi-lobar pneumonia among COVID19 positive BAME patients in comparison to COVID19 positive patients of Caucasian ethnicity.

Furthermore, this study finds that BHS deprivation, which includes an indicator for household overcrowding, is a modulator of severe adverse events requiring admission to ITU. It is already known that household overcrowding is highest among patients of BAME ethnicity [7] and the Intensive Care National Audit and Research Centre (ICNARC) has reported that patients of BAME ethnicity account for 34% of critically ill COVID19 patients nationally despite constituting 14% of the population [20]. Several potential hypotheses have, thus far, been cited in an attempt to explain the increased incidence and admission to ITU among individuals from a BAME background including vitamin D deficiency [21] and genetic predisposition [22] but studies have thus far not explored the potential roles of BHS deprivation or Living Environment deprivation.

In the present study, we believe it to be of interest that, among COVID19 positive patients admitted from regions of highest BHS deprivation, those who died were more likely to have chronic kidney disease, ischaemic heart disease, dementia, higher Charlson Comorbidity Scores and higher Clinical Frailty Scores in comparison to patients who were discharged. This finding suggests that irrespective of BHS deprivation, comorbidities and clinical frailty have a significant role to play in predicting outcomes, death or discharge, among hospitalised COVID19 positive patients. It is of considerable note that comorbidities including ischaemic heart disease, chronic kidney disease, dementia and hypertension significantly increased the risk of death among all COVID19-positive patients. Furthermore, we found it of particular interest that patients presenting with higher Charlson Comorbidity Scores presented with increased severity, CURB 65, which, in turn, increases the risk of mortality. These findings emphasise the modulating role which comorbidities play in predicting outcomes among hospitalised COVID19 positive patients. The UK’s Chief Medical Officer has highlighted that comorbidities and the proportion of patients with two or more medical conditions simultaneously, multi-morbidities, is rising presenting a challenge to the entire medical profession including within acute and long term hospital settings [23]. The literature has well documented the detriment of the comorbidity burden to hospitalised COVID19 patients [24].

It is interesting that despite being younger, presenting with lower Charlson Comorbidity Scores and presenting with lower severity, CURB 65, patients of BAME ethnicity were more likely than Caucasians to present with radiological pneumonia, multi-lobar pneumonia and be admitted to ITU. This picture is likely to be due to having been admitted from regions of highest Living Environment deprivation and highest BHS deprivation but also potentially due to patients of BAME ethnicity presenting later than patients of white ethnicity.

It is intriguing that there was no significance in clinical frailty between BAME and Caucasian groups despite BAME groups presenting younger. Future studies need to explore severity, frailty, comorbidity and deprivation profiles among disaggregated BAME groups in comparison to age and sex matched controls. It is essential that clinical risk tools reflect risk factor profiles to which patients of ethnic minorities are predominantly predisposed to ensure patients from all ethnic groups are triaged to the appropriate level of care. The most commonly used pneumonia severity score in clinical practice, CURB 65, has not thus far been validated for use among COVID19 positive patients nor among individual ethnic minority groups.

There have been several calls for research exploring explanatory factors for the high proportion of deaths in nursing/residential homes in more detail [25,26]. With regards to COVID19 positive patients admitted from nursing/residential homes, this study suggests that there are likely to be a number of modulators of outcomes including: BHS deprivation, comorbidities and clinical frailty. The increased burden of comorbidities among COVID19 positive patients admitted from nursing/residential homes is likely to be a modulator of increased severity, CURB 65, on presentation to hospital.

The present study explores hospitalised COVID19 positive patients within one Birmingham trust. This study does not explore COVID19 positive patients who died in the community or COVID19 positive patients who were not hospitalised or COVID19 positive patients admitted to other hospitals in Birmingham. Future studies need to relate these findings with those of populations from other cities focusing on this level of granularity.

## Conclusion

Household overcrowding deprivation and presentation with radiological multi-lobar pneumonia are potentially important modulators of ITU admission among COVID19 positive patients. Air pollution deprivation and housing quality deprivation are potentially important modulators of presentation with radiological multi-lobar pneumonia. Patients of BAME ethnicity are demographically at increased risk of exposure to household overcrowding deprivation, air pollution deprivation and housing quality deprivation and are more likely to present with radiological multi-lobar pneumonia and be admitted to ITU. However, irrespective of deprivation, comorbidities increase the risk of death among COVID19 positive patients. Consideration of the Charlson Comorbidity Score and the Clinical Frailty Score on admission supports clinicians in stratifying high risk patients and informing the index of suspicion for care. These findings have implications for: supporting front line clinical decisions, disseminating practical advice around applying social distancing messages effectively at the household level and informing wider pandemic strategy.

Future studies should explore the extent to which household overcrowding deprivation, housing quality deprivation and air pollution deprivation, in private and social residences, modulate outcomes among COVID19 positive patients long term and in different cities as this will further inform pandemic strategic planning. Furthermore, future studies should explore effective and validated tools which healthcare professionals can integrate within consultations to acquire a holistic picture of patients’ deprivation risk factors including housing quality deprivation, household crowding deprivation and air pollution deprivation.

## Figures and Tables

**Table 1:** Population demographics (Age, gender and comorbidities among COVID19 positive patients presenting with and without radiological changes within 24 hours of admission).

	All COVID-19 positive patients	COVID-19 positive patients with Radiological changes of Pneumonia	COVID-19 positive patients without Radiological changes of Pneumonia
N	363	294	69
Age, mean (SD)	66.9 (16.7)	66.8 (15.4)	67.0 (21.5)
Gender, n (% of column total)			
Male	217 (59.8)	185 (62.9)	32(46.4)
Female	146 (40.2)	109 (37.1)	37 (53.6)
Ethnicity, n (% of column total)			
Caucasian	231 (63.6)	179 (61.1)	52 (75.4)
Asian/Asian British	87 (21)	75 (25.6)	12 (17.4)
Black/African/Caribbean	26 (7.2)	24 (8.2)	22 (2.9)
Mixed	1 (0.3)	1 (0.3)	0 (0)
Other ethnic groups	10 (2.7)	9 (3.1)	1(1.4)
Unknown	7 (1.9)	5 (1.7)	2 (2.9)
Place of admission, n (% of column)			
Nursing/Residential home	47 (12.9)	38 (12.9)	9 (13.0)
Private residence	315 (86.8)	255 (86.7)	60 (87.0)
Comorbidity, n (% of column)			
Chronic kidney disease	105 (28.9)	82 (27.9)	23 (33.3)
Hypertension	175 (48.2)	150 (51.0)	25 (36.2)
Type 2 Diabetes Mellitus	121 (33.3)	102 (34.7)	19 (27.5)
Dementia	32 (8.8)	23 (7.8)	9 (13.0)
Ischaemic Heart disease	70 (19.3)	55 (19.7)	12 (17.4)
ITU admission, n (% of column)	64 (17.6)	61 (20.7)	3 (4.3)
Discharge, n (% of column)	245 (67.5)	190 (64.6)	55 (79.7)
Mortality, n (% of column)	106 (29.2)	92 (31.3)	14 (20.3)

**Table 2:** Cluster membership profiles: age, gender, ethnicity, place of admission, comorbidities, charlson comorbidity index and clinical frailty score. Cluster membership profiles are of the four emergent clusters from a two-step cluster analysis which was carried out clustering hospitalised COVID19 positive patients by: completed hospitalised episode outcomes and BHS deprivation score.

	All patients	Cluster 1 (Highest BHS deprivation regions and died)	Cluster 2 (All other BHS deprivation regions and died)	Cluster 3 (Highest BHS deprivation regions and discharged)	Cluster 4 (All other BHS deprivation regions and discharged)
n	344	41	64	118	121
Age (Mean (SD) )	67.2 (18.4)	69.9 (14.3)	79.8 (12.0)	59.3 (17.2)	67.5 (15.0)
Gender (n (% within column))					
Male	202 (58.7)	25 (61.0)	39 (60.9)	74 (61.2)	64 (54.2)
Female	142 (41.3)	16 (39.0)	25 (39.1)	47 (38.8)	54 (45.8)
Ethnicity (n (% within column))					
BAME	113 (32.8)	19 (48.7)	14 (22.2)	47 (40.2)	33 (28.2)
Caucasian	223 (64.8)	20 (51.3)	49 (77.8)	70 (59.8)	84 (71.8)
Place of Admission (n (% within column))					
Nursing/Residential home	47 (13.7)	9 (22.0)	20 (31.3)	5 (4.2)	13 (10.7)
Private residence	297 (86.3)	32 (78.0)	44 (68.8)	116 (98.3)	105 (86.8)
Comorbidities (n (% within column))					
Chronic Kidney disease	103 (29.9)	14 (34.1^a,b^)	32 (50.0^a^)	20 (16.5^b^)	37 (31.4)
Dementia	32 (9.3)	9 (22.0^a,b^)	9 (14.1^a^)	5 (4.1^b^)	9 (7.6)
Ischaemic Heart disease	69 (20.1)	12 (30.0^a,b^)	19 (29.7^a^)	16 (13.2^b^)	22 (18.8)
Hypertension	164 (47.7)	21 (52.5^a,b^)	40 (62.5^a^)	51 (42.9)	52 (44.8)
Type 2 Diabetes Mellitus	116 (33.7)	16 (39.0^a,b^)	26 (40.6^a^)	39 (32.2)	35 (29.7)
Charlson comorbidity index (median (IQR))	4.00 (4.00)	6 (5.00)	6 (3.00)	2 (4.00)	4 (4.00)
Clinical frailty score (median (IQR))	4.00 (4.00)	6 (4.00)	6 (3.00)	3 (3.00)	4 (4.00)

a **Note:** Among patients who died, there is no statistical significance in the respective comorbidity profiles between patients admitted from regions of highest BHS deprivation and all other BHS deprivation regions

b Among patients admitted from regions of highest BHS deprivation, there is a statistical significance in the respective comorbidity profiles between patients who died and those who were discharged.

## Data Availability

The datasets used and/or analysed during the current study are included within this manuscript.
